# N-Acetylcysteine and Its Immunomodulatory Properties in Humans and Domesticated Animals

**DOI:** 10.3390/antiox12101867

**Published:** 2023-10-16

**Authors:** Sophie Tieu, Armen Charchoglyan, Lauryn Paulsen, Lauri C. Wagter-Lesperance, Umesh K. Shandilya, Byram W. Bridle, Bonnie A. Mallard, Niel A. Karrow

**Affiliations:** 1 Department of Animal Biosciences, University of Guelph, Guelph, ON N1G 2W1, Canada; stieu@uoguelph.ca (S.T.); ushand@uoguelph.ca (U.K.S.); 2 Department of Pathobiology, University of Guelph, Guelph, ON N1G 2W1, Canada; lpaulsen@uoguelph.ca (L.P.); lwagterl@uoguelph.ca (L.C.W.-L.); bbridle@uoguelph.ca (B.W.B.); bmallard@ovc.uoguelph.ca (B.A.M.); 3 ImmunoCeutica Inc., Cambridge, ON N1T 1N6, Canada; 4 Advanced Analysis Centre, University of Guelph, Guelph, ON N1G 2W1, Canada

**Keywords:** N-acetylcysteine, antioxidant, cytoprotective, immunomodulatory agent, oxidative stress, glutathione

## Abstract

N-acetylcysteine (NAC), an acetylated derivative of the amino acid L-cysteine, has been widely used as a mucolytic agent and antidote for acetaminophen overdose since the 1960s and the 1980s, respectively. NAC possesses antioxidant, cytoprotective, anti-inflammatory, antimicrobial, and mucolytic properties, making it a promising therapeutic agent for a wide range of diseases in both humans and domesticated animals. Oxidative stress and inflammation play a major role in the onset and progression of all these diseases. NAC’s primary role is to replenish glutathione (GSH) stores, the master antioxidant in all tissues; however, it can also reduce levels of pro-inflammatory tumor necrosis factor-alpha (TNF-∝) and interleukins (IL-6 and IL-1β), inhibit the formation of microbial biofilms and destroy biofilms, and break down disulfide bonds between mucin molecules. Many experimental studies have been conducted on the use of NAC to address a wide range of pathological conditions; however, its effectiveness in clinical trials remains limited and studies often have conflicting results. The purpose of this review is to provide a concise overview of promising NAC usages for the treatment of different human and domestic animal disorders.

## 1. Introduction

N-acetylcysteine (NAC) comes from the amino acid L-cysteine and has been a Food and Drug Administration (FDA) approved drug since 1963. It is recognized as the standard treatment for acetaminophen overdose and has been used as a mucolytic drug since the 1960s [1]. NAC has also been used as a supplement for decades and, as such, can be found as an over-the-counter nutritional supplement in countries such as the United States, Canada, and Australia [1]. Therefore, NAC falls into a grey zone with other compounds such as cannabidiol (CBD), but as of August 2022, the FDA has backed away from its hard stance on classifying NAC as a drug and is allowing its sale as a dietary supplement [2]. NAC’s antioxidant and anti-inflammatory properties make it a promising therapeutic agent for conditions in which oxidative stress is involved [1]. Examples of these disorders include diabetes, obesity, cancer, neurological disorders, hypertension, pulmonary, inflammatory bowel, cardiovascular, autoimmune, and infectious diseases in humans, as well as domesticated animal weaning disorders, respiratory disease, diarrhea, endometritis, and mastitis [3,4,5,6,7,8,9,10].

While there has been an increasing interest in the use of NAC for a wide range of pathological conditions over the past few decades, various human clinical studies have reported conflicting results, and many other studies were performed in vitro. Therefore, more clinical studies are required to address these conflicting results and to support the purported therapeutic roles of NAC in treating different pathological conditions.

Various animal studies involving the use of NAC have also been performed, especially with its use in treating intestinal inflammation and related disorders that can be caused by anti-nutritional factors (e.g., *β*-conglycinin, a vicilin storage protein of soybeans), the process of weaning, and consumption of mycotoxins [10,11,12]. Many studies have shown the protective effects of NAC for neonatal animals immune challenged with bacterial lipopolysaccharide (LPS) endotoxin, particularly studies involving piglets [13,14]. NAC administration has consistently been shown to increase daily body weight gain and alleviate LPS-mediated growth depression. Furthermore, the porcine model of ulcerative colitis (UC) can be used to support NAC usage for humans. For example, in a study conducted by Wang et al. (2013), the piglet model of UC demonstrated that administration of NAC was able to reduce colon histopathology score and ameliorate UC histological abnormalities [15].

Despite the numerous NAC studies and its increasing popularity, the mechanisms of action (MOA) by which NAC exerts its antioxidant and cytoprotective properties remain unclear [1,16]. It is often assumed that the effects conferred by NAC are due to it acting as a scavenger of reactive oxygen species (ROS), a precursor for glutathione (GSH) biosynthesis, and a disulfide reductant [16,17]. However, these three major narratives can only explain the effects of NAC under specific circumstances [16,17]. Recently, an alternative MOA was proposed that may explain the effects attributed to NAC: the conversion of NAC into hydrogen sulfide and sulfane sulfur species, which are known to possess antioxidant and cytoprotectant properties [16,17]. Thus, the purpose of this review is to provide an overview of the therapeutic uses of NAC in both humans and domesticated species, with a particular focus on weaning disorders, as well as an overview of its MOA.

### NAC Formulations

The most common and well-known formulation of NAC in the United States is Mucomyst™, which is commonly administered orally for the treatment of acetaminophen toxicity [18]. PharmaNAC^®^ (BioAdvantex Pharma Inc., Mississauga, ON, Canada) is another common oral formulation and is the only effervescent preparation of its kind available in North America [19]. Due to its disagreeable flavor, NAC is often mixed with fruit juice or a soft drink prior to consumption. In Europe, NAC is available in pills, capsules, and a variety of effervescent “fizzy tab” formulations [18].

## 2. Safety Profile of NAC

The safety of NAC has been well-established through numerous pharmacological studies [17]. Toxicity is rare and dependent on the route of NAC administration and dosage [1]. NAC can be administered orally, intravenously, or intranasally [1]. When orally administered, NAC undergoes rapid intestinal absorption and is subsequently metabolized by the liver [1]. The cysteine released during NAC metabolism is utilized for glutathione (GSH) synthesis, which is vital for immune function and tissue repair [1]. NAC’s bioavailability following oral administration is less than 10%. Therefore, only a small portion of intact NAC reaches the plasma and tissue [1]. It has been suggested that the low bioavailability of oral NAC may be due to first-pass metabolism in the small intestine rather than incomplete absorption [20]. NAC can be found intact, reduced, or in various oxidized forms in the plasma following oral intake [1,20], and individual variation in these NAC metabolites occurs, which is likely due to natural variations in human metabolism and the intestinal microbiota. Comparatively, intravenous or nasal administration allows for rapid delivery of high concentrations of NAC to the circulation as it bypasses the first-pass intestinal and hepatic metabolic pathway [1]. Considering that NAC bioavailability is influenced by first-pass metabolism, it will be important to address potential gender and age effects, which are known to affect the first-pass metabolism [21,22]. Therefore, an optimal way to maintain a proper therapeutic serum level of NAC after intake should include monitoring NAC metabolite concentrations in the blood to maximize both the efficacy of treatment and patient safety.

## 3. Transportation of NAC

NAC is transported into cells through a complex process involving active transport mechanisms. Within cells, NAC is primarily absorbed and utilized in the cytoplasm, where it is converted into cysteine. NAC is observed to have a slower rate of cellular absorption compared to cysteine [23]. This delayed uptake is attributed to the hindrance caused by the N-acetyl group, which affects both passive and active transportation across the cell’s plasma membrane. Passive transport of NAC is particularly disadvantaged due to its negative charge at physiological pH, unlike Cys, which is a zwitterion with no net charge, resulting in reduced membrane permeability [24]. Remarkably, modifications neutralizing the charge on NAC’s carboxyl group, such as amidation or esterification, have been shown to significantly enhance its cellular uptake, as previously reported [25,26]. Despite these findings, there is currently no substantial evidence suggesting the existence of active membrane transport systems specific to NAC. ASCT1, the canonical importer of reduced Cys, does not transport negatively charged amino acids, including NAC [27]. While there have been reports implicating anion exchanger 1 (AE1) as a potential facilitator of NAC uptake into erythrocytes, this remains an area requiring further investigation, as pointed out by Raftos et al. (2007) [23]. Once absorbed, NAC must undergo deacetylation to yield cysteine. Aminoacylase 1 is believed to be the primary enzyme responsible for deacetylating NAC in this process.

### 3.1. Adverse Reactions

Adverse effects following administration of NAC range from mild to severe and are dependent on the formulation, concentration, and route of administration [1,28] (Table 1). Intravenous NAC and oral NAC are commonly associated with minimal side-effects [1], such as symptoms of nausea, vomiting, pruritus, and erythema [28,29]. The frequency of NAC side-effects following intravenous administration is significantly higher compared to oral NAC [18]. Inhaled NAC can result in adverse reactions such as bacterial pneumonia, cough, sore throat, and drug-induced pneumonitis, but coughing is the most prevalent [1,30]. Anaphylactic reactions resulting from NAC are rare and mild or moderate depending on the concentration [31]; most are typically attributed to intravenous administration due to the transient but significant increase in NAC plasma levels [18]. Existing data suggests that there is a direct relationship between anaphylactoid reactions and serum NAC concentration [31]. Symptoms of anaphylactoid reactions include flushing, pruritus, angioedema, bronchospasm, and hypotension [32]. These anaphylactoid reactions rapidly subside following the discontinuation of NAC administration or lowering the rate of intravenous administration [18].

### 3.2. NAC Dosing and Pharmacokinetics

NAC is commonly taken orally at doses of 600–1200 mg daily to treat specific conditions, or even as a dietary supplement. Administration of oral NAC at doses as high as 8000 mg/day is well-tolerated with no clinically significant adverse reactions [33]. NAC has a half-life of 5.58 h after intravenous administration [20]. Its half-life following oral administration is 6.25 h, reaching a maximum plasma concentration (Cmax) approximately 1 to 2 h post administration [20].

## 4. Therapeutic Uses of NAC in Animals

While studies have been conducted on the therapeutic uses of NAC in livestock and companion animals, in addition to its use as an antidote for acetaminophen poisoning in dogs and cats, limited pharmacokinetic data on NAC exists in the literature for other species. Among the limited pharmacokinetic data available, only two studies discussed the pharmacokinetics of NAC in chickens and healthy cats. In the study conducted by Buur et al. (2013), the half-life of NAC following IV and oral administration (100 mg/kg) in healthy cats was found to be 0.78 ± 0.16 h and 1.34 ± 0.24 h, respectively, and the bioavailability of NAC following oral administration was 19.3 ± 4.4% [34]. The pharmacokinetics of NAC found in the six cats used in this study differs from the values reported for humans; thus extrapolating dosages from human medicine may lead to underdosing cats with acute disease. This study has limitations, however, given its small population size and the assumption that the pharmacokinetics of NAC in diseased cats are similar to that of healthy cats. In the study conducted by Petkova and Milanova (2021), NAC was found to reach a maximum plasma concentration of 2.26 ± 0.91 g/mL 2.47 ± 0.45 h following oral administration of 100 mg/kg in healthy broilers [35]. NAC’s half-life was found to be 1.04 ± 0.53 h. The authors also did not find any significant difference between NAC’s pharmacokinetics in healthy broilers versus Mycoplasma gallisepticum-infected broilers.

## 5. Therapeutic Uses of NAC in Humans

NAC is one of the most used antioxidants in clinical practice to date [16,17,36]. There has been a growing interest over the past few decades to use NAC as a potential treatment for a wide range of diseases and disorders in which oxidative stress is suspected to play a role [17,37], including respiratory, cardiovascular, neurodegenerative, liver, kidney, gastrointestinal, and infectious diseases [1].

**Table 1:** N-acetylcysteine adverse events.

Study	Study Type	Study Size	NAC Treatment	Type and Incidence of Adverse Events following NAC Treatment		
					Moderate	Severe
[38]	Double-blinded, placebo-controlled, crossover design	17 healthy individuals (age 30 ± 2 years)	9 mg/kg NAC capsule	Upset stomach (2), nausea (1), stomach/intestinal gas (1), cough (1)		
			18 mg/kg NAC capsule	Upset stomach (1), nausea (1), stomach/intestinal gas (2), sleepiness (2), metallic taste (1)		
			35 mg/kg oral NAC solution	Upset stomach (2), stomach/intestinal gas (2), sleepiness (1), metallic taste (1)		
			70 mg/kg oral NAC solution	Upset stomach (2), stomach/intestinal gas (1), sleepiness (3), metallic taste (3), light-headedness (1), cough (1)	Upset stomach (1),	Stomach/intestinal gas (1)
			140 mg/kg oral NAC solution	Upset stomach (5), nausea (3), stomach/intestinal gas (2), sleepiness (2), metallic taste (4), light-headedness (1)	Upset stomach (1), stomach/intestinal gas (4)	
[39]	Randomized, placebo-controlled study	28 individuals (≤75 years)	20 mg/min intravenous NAC for first hour, then 10 mg/min for next 23 h; total dose of 15 g over 24 h	Haemorrhage (3), headache (4)		Transient episode of extreme sinus bradycardia (1)
[40]	Randomized, placebo-controlled study	4 healthy individuals (age 35 ± 3 years)	150 mg/kg intravenous	Transient skin flushing (2), pruritus (2), nausea (2)		
[41]	Double-blind, randomized trial	65 chronic bronchitis patients	4 puffs of NAC (1 mg/puff) two times daily	Coughing (4), Dyspnoea (7)		

### 5.1. Liver Diseases

Acute and chronic liver diseases are highly prevalent worldwide, accounting for approximately 2 million deaths per year [42]. Oxidative stress plays a crucial role in the initiation and progression of liver diseases due to its participation and stimulation in the liver’s fibrogenic response [1]. Numerous clinical and experimental studies have been conducted on the use and efficacy of NAC in modulating inflammation and oxidative stress caused or propagated by liver diseases, including acetaminophen (paracetamol) poisoning, acute liver failure, and non-alcoholic fatty liver (NAFLD) and alcoholic liver diseases (see Table 2).

#### 5.1.1. Acute Acetaminophen Overdose

Acetaminophen is a commonly used over-the-counter analgesic and antipyretic medication as it is relatively safe and effective in treating mild-to-moderate pain and fever at appropriate doses [28,43]. However, when taken by adults at doses of 10–15 g (single or repeated) over a 24 h period, which is 3–5 times the manufacturer’s recommended dose for over-the-counter use, acetaminophen has direct hepatotoxic potential and can cause acute liver injury and death from acute liver failure [28,43]. Following ingestion, acetaminophen is rapidly absorbed and transported to the liver to undergo first-pass metabolism [28]. N-acetyl-p-benzoquinonimine (NAPQI), a cytochrome P450-derived metabolic by-product of acetaminophen first-pass metabolism is cytotoxic and genotoxic [28]. Normally, when acetaminophen is taken at low doses, GSH can detoxify NAPQI by conjugating it, which facilitates its elimination from the body [28]. However, acetaminophen toxicity leads to the formation and accumulation of excessive amounts of NAPQI, the depletion of GSH stores, oxidative stress, and mitochondrial dysfunction resulting in the depletion of adenosine triphosphate (ATP) stores [44]. Evidence suggests that NAPQI is capable of binding to several cellular proteins, particularly mitochondrial proteins [44]. This binding to mitochondrial proteins in the context of GSH depletion is of great significance as it results in the depletion of endogenous antioxidant functions and alters the ∝-subunit of the mitochondrial ATP-synthase, thereby hindering the production of ATP [44]. NAC functions to replenish hepatic GSH stores and provide a larger supply of oxygen to the injured liver [1]. Administration of NAC during acetaminophen overdose acts to rapidly increase GSH synthesis in the liver and reduce mitochondrial protein binding [28]. The amount of NAC required to counteract acetaminophen toxicity is determined by plotting the concentration of acetaminophen in plasma against the time post-overdose on a nomogram [28]. Initiating NAC treatment within 8 h of overdosing minimizes the risk of hepatocellular damage as the effectiveness of NAC treatment is negatively correlated with the time post-overdose [28]. Reports have shown that oral NAC is just as effective as intravenous NAC in treating acetaminophen toxicity within 10 h of overdosing [28]. However, it is important to note that the absorption of orally administered NAC will be hindered when administered following the use of activated charcoal, which is commonly used to mitigate acetaminophen toxicity; as such, the preferred route of administration is intravenous NAC [28].

#### 5.1.2. Non-Acetaminophen-Induced Acute Liver Failure

Etiological agents of non-acetaminophen-induced acute liver failure can include viruses, drugs, toxins, herbal and traditional medicines, and autoimmune-mediated conditions [45]. Immediate intravenous administration of NAC in cases of non-acetaminophen-induced liver failure has been found to reduce mortality, encephalopathy, hospitalization, admission to the ICU, organ failure, and the need for liver transplantation [1].

#### 5.1.3. Non-Alcoholic Fatty Liver Disease (NAFLD)

NAFLD refers to a condition in which an excessive amount of fat is stored in hepatic cells [46]. Under normal physiological conditions, the liver stores small amounts of energy in the form of the carbohydrate glycogen [46]. Therefore, a healthy liver should contain little to no fat droplets. Livers that contain fat droplets in more than 5% of hepatic cells are abnormal or pathological [46]. The accumulation of fat in the hepatocytes of NAFLD patients is generally due to a combination of excessive calorie intake and a sedentary lifestyle [46]. However, diabetic patients, particularly type 2 diabetics, and those with abnormal levels of blood lipids or hypertension are also at risk of developing NAFLD [46]. Regardless of the etiological factor(s) leading to the onset of NAFLD, the increased flow of free fatty acids to the liver concurrently increases oxidative stress and suppresses hepatic intracellular antioxidant activity [1].

Experimental studies conducted on the use of NAC in NAFLD patients have demonstrated that NAC can block the accumulation of hepatic lipids and reduce the pro-inflammatory cytokines IL-6, IL-1β, and TNF-∝ and the upstream transcription factor NF-κB [47], which plays a critical role in initiating the inflammatory cascade and immune response related to oxidative stress. NAC has been purported to possess anti-inflammatory properties that include inhibiting activation and translocation of NF-κB, which results in decreased production of TNF-∝, IL-1β, and IL-6 [1]. In a human study conducted by Khoshbaten et al. (2010), it was discovered that oral administration of 600 mg of NAC per 12 h over a 3-month period decreased serum levels of alanine transaminase (ALT) as compared to the group receiving vitamin C [48]. ALT is an enzyme that is predominantly produced by the liver and is commonly used as a biomarker of hepatic inflammation and damage [49].

### 5.2. Pulmonary Diseases

Inflammation and high levels of oxidative stress coupled with low levels of endogenous antioxidants such as GSH play a critical role in the pathogenesis and progression of pulmonary diseases [1]. As an antioxidant, anti-inflammatory, and mucolytic agent, NAC appears to be a promising therapeutic agent in the treatment of various pulmonary diseases including chronic obstructive pulmonary disease (COPD), cystic fibrosis (CF), and idiopathic pulmonary fibrosis (IPF) [1] and, most recently, COVID-19 [50] (see Table 2). As a therapeutic agent, NAC is commonly administered orally in the tablet form of 600–1200 mg up to three times a day to treat pulmonary diseases [1]. Studies have shown that dosages of NAC up to 3000 mg/day continue to remain safe and are well-tolerated [1].

#### 5.2.1. Cystic Fibrosis (CF)

CF is a genetic disorder affecting multiple organ systems including the lungs and upper airways, pancreas, liver, intestine, and reproductive organs [51]. The lungs of CF patients produce prolific amounts of viscous mucus, which is difficult to clear. This thick mucus increases susceptibility to recurring chronic infections due to poor expectoration [28]. NAC has been extensively used in the treatment of CF to help improve lung function and eliminate mucus due to its mucolytic properties [1], which are attributed to its cysteine residues that break down the sulfhydryl bridges between glycoproteins in mucus, thereby reducing mucus viscosity [28].

Excessive neutrophil-mediated inflammation in the airways is another key characteristic of CF and is believed to be a cause of lung damage and dysfunction [52]. This uncontrolled inflammation leads to overexposure to reactive oxygen species (ROS) derived from bacteria and/or the activated neutrophils, which in turn further amplifies the inflammation [52] and damages tissues. In addition to being a mucolytic agent, NAC’s antioxidant properties may prove to be useful in controlling oxidative stress and excessive inflammation in the airways of CF patients [52]. In a study conducted by Dauletbaev et al. (2009), it was found that a 12-week therapy with a high dose of NAC (2800 mg/day) increased extracellular GSH in the sputum of CF patients; however, NAC treatment did not appear to alter any clinical or inflammatory parameters [52].

#### 5.2.2. Chronic Obstructive Pulmonary Disease (COPD)

COPD refers to a group of chronic progressive lung diseases that cause airflow obstruction and breathing-related issues [53]. COPD is predominantly caused by cigarette smoking; however, long-term exposure to other lung irritants, such as second-hand smoke, air pollution, chemicals, work-related fumes, and toxic substances can also lead to COPD [53]. While many diseases fall under the umbrella of COPD, chronic bronchitis and emphysema are the two most common types.

The presence of numerous free radicals and oxidants in cigarette smoke contributes to lung inflammation through the induction of oxidative stress. During severe COPD, oxidative stress has been found to be exacerbated and GSH levels further depleted; GSH homeostasis is one of the most important antioxidant defense systems in lung cells [54]. Although inhaled bronchodilators and corticosteroids remain the main treatment for COPD, oxidative stress and inflammation represent promising therapeutic targets for treatment due to their key role in the pathogenesis of COPD [54]. In a study conducted by Messier et al. (2013), NAC was shown to provide protection against injury to murine alveolar type 2 (ATII) cells and lung tissue induced by cigarette smoke [55]. These in vivo and in vitro studies were carried out with mice lacking the nuclear factor erythroid 2-related factor-2 (*Nrf2*), whose gene product is a redox-sensitive transcription factor that is crucial to the regulation of the antioxidant defense system [54,55]. NAC’s ability to act as a direct scavenger of free radicals in an Nrf2-independent manner is of considerable importance as Nrf2-dependent endogenous antioxidants are often reduced in COPD patients [54]. In a randomized, placebo-controlled trial conducted by Kasielski and Nowak (2001), in which NAC was administered long-term (12 months), it was found that treatment with 600 mg/day of NAC significantly reduced the concentration of H_2_O_2_ in the expired breath condensate of stable COPD patients; these patients showed a progressive decrease from baseline H_2_O_2_ concentrations, reaching statistical significance after 6 months of treatment [56]. After 9 and 12 months of treatment, the concentration of H_2_O_2_ in the expired breath condensate was 2.3- and 2.6-fold lower, respectively, than in COPD patients in the placebo group. Based on the current data, the Global Initiative for Chronic Obstructive Lung Disease has acknowledged that NAC can be used as an adjunct therapy to help reduce the risk of acute exacerbation of COPD [1].

### 5.3. Infectious Diseases

NAC’s ability to attenuate mediators of oxidative stress and inflammation also makes it a promising treatment or adjuvant for various infectious diseases, such as influenza and COVID-19 (see Table 2).

#### 5.3.1. Influenza

Influenza, also commonly referred to as “the flu”, is a contagious respiratory infection caused by various influenza viruses that infect the nose, throat, and lungs [57]. The current standard treatment of severe flu includes early antiviral therapy with a neuraminidase inhibitor, which is associated with improved outcomes in hospitalized seasonal influenza patients [58]. However, a significant number of deaths (~25% mortality) still occur in ICU patients with influenza A (H1N1) despite the use of antivirals [58].

Patients with severe influenza A virus infection (H1N1, H5N1, and H7N9) were found to have high levels of circulating pro-inflammatory cytokines [58]. Due to the significant role that inflammation plays in the pathogenesis of seasonal flu, various studies have been conducted to assess the effectiveness of NAC for the treatment of influenza pneumonia. NAC was found to have indirect anti-viral effects, along with the ability to decrease pro-inflammatory cytokine levels and exert anti-apoptotic activities [59]. NAC can help alleviate the symptoms of influenza, primarily by addressing different mechanisms associated with the infection. However, direct impacts could be because of a reduction in viral load [60]. It has been proposed that NAC might interfere with the ability of the influenza virus to replicate and spread within the body. In human trials, for example, NAC significantly lowered the occurrence of clinically apparent H1N1 influenza disease [61]. Additionally, in cell culture experiments, NAC protected against H3N2 influenza virus-induced oxidative stress, cell death, the expression of inflammatory genes, and NF-κB activity [62,63]. However, this aspect is still under investigation, and more research is needed to confirm its antiviral properties. High doses of NAC used as an adjuvant treatment in influenza pneumonia patients have also been shown to reduce influenza symptomatology and to improve cell-mediated immunity, which is important for fighting viral infections [61]. NAC’s immunomodulatory properties are likely due to its ability to inhibit the activation of oxidant-sensitive pathways, including the NF-κB and p38 mitogen-activated protein kinase (MAPK) signaling pathways [60]. Indeed, cytokine and chemokine levels were significantly reduced following in vivo NAC treatment [60]. Lower numbers of infiltrating macrophages, lymphocytes and neutrophils, and myeloperoxidase (MPO) activity, were also found in influenza-infected lungs following administration (intraperitoneal injection) of NAC in mice [64]. Furthermore, the increased proliferation of influenza-specific lymphocytes and the effector function of cytotoxic lymphocytes were found to be positively correlated with NAC treatment [65]. However, it is important to note that NAC efficacy is dependent on the strain of influenza virus, particularly influenza A virus [59]. It appears that NAC’s antioxidant and immunomodulatory properties are more efficacious for highly pathogenic influenza A strains in comparison to low pathogenic influenza A strains [60,66]. While the reasoning behind this strain-dependent variation in efficacy is not yet well understood, it is thought that the differences in terms of how the NF-κB pathway is activated in highly pathogenic versus low pathogenic influenza strains may provide an explanation [59].

#### 5.3.2. COVID-19

COVID-19 deaths are mainly attributed to the acute respiratory distress syndrome (ARDS) associated with SARS-CoV-2 infection, particularly in the elderly and those with co-morbidities [67,68]. There is increasing evidence to indicate that excessive immune activation, and the resulting cytokine storm, are the cause of COVID-19-associated lung injury [67]. This excessive immune activation can be the result of an imbalance in redox homeostasis of which prolonged oxidative stress due to inflammation, increased ROS production, and decreased GSH levels are important factors [67].

NAC has long since been used as an off-label antioxidant; however, there is increasing evidence from both preclinical and clinical studies that NAC can attenuate immune activation and cytokine release, which may be relevant to COVID-19 [50]. In a study conducted by Ungheri et al. (2000), NAC was found to significantly decrease the mortality of influenza-infected mice by reducing the production of ROS and cytokines, such as TNF-∝ and IL-6 [69]. Furthermore, the addition of NAC to the standard treatment protocol for many acute respiratory conditions including influenza, community-acquired pneumonia (CAP), ARDS, and ventilator-associated pneumonia (VAP) was found to decrease the severity of disease by mainly attenuating the immune activation [67].

NAC could be used as a potential therapeutic agent in the treatment of COVID-19 by supporting T-cell responses and modulating inflammation [70]. It might also have a potential application to neutralize the toxicity of the SARS-CoV-2 spike protein both after COVID-19 infection and mRNA-based injection. Almost all SARS-CoV-2 variants have conserved cysteine residues in the spike protein forming disulfide bonds. Upon in silico exposure to NAC, these SARS-CoV-2 cysteine residues conjugate covalently with NAC, which results in perturbation of stereo-specific orientations of spike protein and consequent weakening in the binding affinity of spike protein with ACE2 receptor [71].

Given the major roles that ROS and the cytokine storm play in the pathogenesis of COVID-19, the use of NAC to treat COVID-19 has been proposed [67]. In a small case study (10 cases) conducted by Ibrahim et al. (2020), it was found that the acute-phase protein serum C-reactive protein (CRP) and ferritin levels were decreased in all severe COVID-19 hospitalized patients following twice-daily intravenous administration of 600 mg of NAC [72]; elevated CRP and ferritin levels are indicative of uncontrolled inflammation [73]. Additionally, a significant improvement in liver function and reduced oxygen requirement was also observed in all ten patients enrolled in the Ibrahim et al. (2020) study following the administration of NAC; nine of these patients that previously required extracorporeal membrane oxygenation (ECMO) showed significant improvement and their ECMO treatment was discontinued after NAC therapy [72]. It is worth noting, considering the recent publication on the potential of immune tolerance induction via IgG4 following COVID-19 booster vaccinations [74], that as early as 1997, De Flora et al. showed that oral administration of NAC (600 mg 2 times/day for 6 months) significantly improved cell-mediated immunity to influenza, shifting the response from tolerogenic to activation in seniors [61]. Although immunological tolerance or energy is appropriate under certain circumstances, such as eliminating reaction to self or food antigens, it can also be inappropriate when it eliminates or limits protective responses to pathogenic agents. The potential to utilize NAC to modulate immunological tolerance is worthy of further investigation.

In summary, clinical studies, as detailed in Table 2, highlight several key findings regarding N-acetylcysteine (NAC). Firstly, NAC stands as a well-established and effective treatment for acetaminophen (paracetamol) overdose. Consistent evidence demonstrates that administering NAC promptly after an overdose can prevent or mitigate liver damage, leading to improved patient outcomes. Secondly, research on NAC’s efficacy in chronic obstructive pulmonary disease (COPD) has yielded mixed results. While some studies suggest that NAC may reduce exacerbation frequency and enhance lung function in COPD patients, others have not observed significant benefits. Additionally, NAC serves as a mucolytic agent, aiding individuals with respiratory conditions like cystic fibrosis and chronic bronchitis in clearing mucus from their airways. Clinical investigations affirm its effectiveness in facilitating mucus clearance and enhancing lung function in such cases. Moreover, there is emerging evidence indicating that NAC may reduce oxidative stress, enhance endothelial function, and decrease inflammation, all of which are pertinent to cardiovascular health. Nevertheless, further research is needed to comprehensively understand its impact on cardiovascular outcomes. Furthermore, NAC has been the subject of study for its potential hepatoprotective properties, revealing promising outcomes by lowering liver enzyme levels and enhancing liver function. Lastly, NAC may play a role in alleviating influenza symptoms and aiding recovery, primarily due to its antioxidant and immunomodulatory characteristics.

## 6. Autoimmune Diseases

Autoimmune diseases encompass a wide variety of illnesses and occur when self-constituents are attacked by a hyperactive immune system [75,76]. Due to a loss of immunological self-tolerance, immune cells begin to attack self-molecules manifesting as an autoimmune response [75]. Inflammatory bowel diseases (IBD) are autoimmune diseases that cause chronic inflammation of the gastrointestinal tract, and they are categorized into two main types, UC and Crohn’s diseases [76]. Autoimmune diseases are becoming increasingly prevalent, and their corresponding treatments can result in further immunosuppression, which can lead to systemic infections that potentially cause death [75].

More natural treatment remedies, such as NAC, have been explored for treating IBD [77]. Studies conducted by Ebrahimi et al. (2008) measured certain biomarkers in colon cells in a mouse model to examine the effects of NAC on IBD. NAC was administered at varying amounts (106, 160, and 240 mg/kg) over the course of four days after the induction of colitis, and it was determined that NAC was able to attenuate lipid peroxides, the cytokine TNF-⍺, and nitric oxides [77]. The researchers concluded that cellular biomarkers for IBD improved with the use of moderate to high doses of NAC [77]. Additionally, research on human UC by Shirazi et al. (2021) examined NAC as an antioxidant agent for treating flare-ups of the illness [78]. In a double-blind controlled clinical trial, patients received 800 mg of NAC or placebo over the course of 16 weeks [78]. The results of the study found significant differences between the two treatment groups, where the NAC-treated patients had fewer incidences of endoscopic relapse compared to the placebo group [78]. Additionally, serum CRP levels, mean fecal calprotectin, and the serum erythrocyte sedimentation rate were lower in the NAC group than in the placebo group [78]. These findings elucidated the positive effects that NAC has on the treatment of UC [78]. Further studies regarding the protective effects of NAC in treating IBD should be explored in humans seeing as it was proven in mice that NAC can improve cellular biomarkers of IBD disease and elicit positive effects in the treatment of UC within humans [78].

## 7. Cardiovascular Diseases

Recently, the role of NAC in cardiovascular diseases has also received wide attention from the research community. It has been found that NAC can effectively inhibit myocardial cell apoptosis caused by ischemia-reperfusion injury (IRI) and improve cardiac function [79]. NAC may have an indirect effect on the levels of low-density lipoprotein (LDL) and oxidized LDL, primarily through its antioxidant and anti-inflammatory properties. By increasing GTH levels, NAC helps reduce oxidative stress and the formation of reactive oxygen species (ROS). High oxidative stress can lead to the oxidation of LDL cholesterol, transforming it into oxidized LDL, which is more atherogenic. By reducing oxidative stress, NAC may help inhibit the formation of oxidized LDL [80,81]. It is also reported that NAC may improve the function of endothelial cells lining blood vessels [82]. When the endothelium is healthy, it produces nitric oxide, which helps to relax blood vessels and regulate blood flow. Improved endothelial function can contribute to a better balance of LDL and high-density lipoprotein (HDL) cholesterol. NAC may have these potential benefits, but its impact on LDL and oxidized LDL levels may vary among individuals. The research in this area is still evolving, and the effects of NAC on cholesterol and lipoprotein profiles may be influenced by various factors not discussed here.

## 8. Chronic Conditions

### 8.1. Atopic Dermatitis

Atopic dermatitis is a chronic relapsing inflammatory skin disease that is associated with epidermal barrier dysfunction [83]. The most common symptoms patients experience are chronic pruritus and eczematous lesions, which can adversely impact the quality of life for individuals afflicted by this disease [84]. In more recent years, drug therapies targeting the type 2 antibody-mediated immune response have shown a decrease in signs and symptoms of atopic dermatitis; however, the exorbitant expenses of these pharmaceutical agents may restrict their prolonged usage [84]. The use of NAC, which is relatively safe and inexpensive, as an alternative therapeutic agent to these more expensive drugs shows promising results for treating atopic dermatitis [83,85].

Clinical effects of topical NAC for treating dermatitis have proven to increase skin hydration in patients suffering from atopic dermatitis [83]. By measuring skin hydration and trans-epidermal water loss, it was determined that NAC had the ability to reduce oxidative stress and allowed for the restoration of adhesion molecules involved in forming the skin barrier, leading to increased skin hydration in patients with atopic dermatitis [83]. Future research is required to determine the molecular pathways NAC plays in restoring the expression of these adhesion molecules and if NAC would be a suitable candidate for treating other skin ailments such as psoriasis [83].

### 8.2. Diabetes Mellitus

The prevalence of type 2 diabetes, a multifactorial disease characterized by progressive deterioration of insulin secretion and action, is on the rise and accounts for more than 90% of individuals diagnosed with diabetes [86]. Diabetes is primarily due to insulin resistance, and while the pathogenesis of insulin resistance is not yet clear, oxidative stress, innate immune system activation, and abnormal lipid and/or energy metabolism are considered to play key roles [86].

Several clinical studies have been conducted on the use of NAC as a potential therapeutic agent for insulin resistance and type 2 diabetes [28] (Table 1). In a study conducted by Ribeiro et al. (2011), it was discovered that NAC exhibited beneficial modulatory action on oxidative stress biomarkers in alloxan-induced diabetic rats [87]. In another study conducted by Kaneto et al. (2001), NAC was shown to exert protective effects on the pancreatic β cells of diabetic db/db mice [88]. In this study, prior to NAC treatment, hyperglycemia episodes in mice led to decreased insulin content and insulin gene expression. However, following treatment with NAC, insulin content and insulin mRNA expression were found to be preserved, and the binding of the nuclear factor pancreatic-duodenal homeobox-1 (PDX-1) to insulin was also restored. Furthermore, studies involving diabetic patients demonstrated that the administration of intravenous NAC during hyperglycemic clamp, which measures insulin secretion and pancreatic-cell function, was shown to improve insulin sensitivity and increase peripheral glucose uptake [89].

**Table 2:** N-acetylcysteine clinical studies.

Disease	Study Type	Study Phase	Dose	Treatment Duration	Administration Routes
***Liver diseases***					
*Acute acetaminophen overdose*	Interventional (Clinical Trial) NCT03679442	Phase 1	Dose corresponding to the clinical treatment guidelines for acetaminophen overdosed patients	16 h	Intravenous
*Non-alcoholic fatty liver disease*	Interventional (Clinical Trial) NCT02117700	Phase 2	600 mg twice/ day	16 weeks	Oral
***Pulmonary diseases***					
*Cystic fibrosis*	Interventional (Clinical Trial) NCT00809094	Phase 2	900 mg twice/day	24 weeks	Oral
*Chronic Obstructive Pulmonary Disease*	Interventional (Clinical Trial) NCT01136239	Phase 4	600 mg twice/ day	One year	Oral
	Interventional (Clinical Trial) NCT00969904	Phase 4	600 mg twice/ day	12 weeks	Oral
	Interventional (Clinical Trial) NCT03388853	Phase 4	1200 mg once daily	4 weeks	Oral
	Interventional (Clinical Trial) NCT02579772	Phase 4	600 mg three times/ day for 4 days prior to experimental procedures and 600 mg on the day of the experiment	4 days	Oral
	Interventional (Clinical Trial) NCT00184977	Phase 4	600 mg once daily	3 years	Oral
***Infectious Diseases***					
*Influenza*	Interventional (Clinical Trial) NCT03900988	Phase 4	100 mg/kg daily as a continuous IV infusion over 24 h	28 days	Intravenous
*COVID-19*	Interventional (Clinical Trial) NCT04374461	Phase 2	6 g/day	Patients will receive treatment for a max of 3 weeks	Intravenous
	Interventional (Clinical Trial) NCT04928495	Phase 3	1800 mg once daily	10 days	Oral
	Interventional (Clinical Trial) NCT04900129	Phase 1	1.2 g twice/day	One month	Inhalation
	Interventional (Clinical Trial) NCT04792021	Phase 3	600 mg/day	Two weeks/ until hospital discharge or death	Oral
	Interventional (Clinical Trial) NCT04419025	Phase 2	**Inpatients**: 25 mg/kg (rounded up to the nearest 600 mg) every 4 h until discharge and then 1200 mg twice daily × 1 week post-discharge **Outpatients**: 2400 mg × 1 then 1200 mg twice daily × 2 weeks	**Inpatients**: until 1 week post-discharge **Outpatients**: 15 days	Oral
	Interventional (Clinical Trial) NCT04455243	Phase 3	150 mg/kg every 12 h diluted in 200 mL diluent (D5%, NS)	14 days	Oral or intravenous
***Diabetes Mellitus***	Interventional (Clinical Trial) NCT02206152	Phase 1 and 2	150 mg/kg loading dose over the first hour and then follow that with a 50 mg/kg maintenance dose infused over the next 4 h during a controlled hyperinsulinemic hypoglycemic insulin clamp	Two 2-day treatments separated by 8 weeks	Intravenous
	Interventional (Clinical Trial) NCT01394510	N/A	600 mg twice daily × 2 weeks, then 1200 mg twice daily × 2 weeks	4 weeks	Oral
	Interventional (Clinical Trial) NCT04531163	Phase 2 and 3	1200 mg/day	2 months	Oral
	Interventional (Clinical Trial) NCT00556465	Phase 2 and 3	600 mg twice/ day	3 months	Oral

## 9. Use of NAC in Domesticated Animal Health and Production

Intestinal integrity is essential to normal physiological function as it is critically involved in nutrition, metabolism, and whole-body homeostasis [10]. Damage to the mucosal epithelium impairs nutrient absorption and compromises immunity. Consequently, this reduces animal growth performance and compromises animal health [10]. Animal stressors, such as early weaning and infection, result in injury and dysfunction of the intestinal mucosal barrier [10], which serves as the first line of defense against endogenous and exogenous microbes and their toxins [10]. While the use of NAC for domesticated animals is not widespread, or common, there is substantial evidence to support its use, particularly during the weaning period to help increase average daily gain, increase food intake, and improve growth performance of neonates. Many nutrients have recently been reported to improve immune function, particularly during stress [90], and, therefore, it is not surprising that NAC may also be considered an immunoceutical because of its immunomodulatory properties.

## 10. Swine

There is increasing evidence that dietary supplementation of NAC may improve the intestinal morphology and function of livestock species, particularly piglets [10,91]. Indeed, recent studies have found that administration of NAC reduces inflammation, alleviates oxidative stress, improves energy status, and ameliorates intestinal tissue damage in piglets that were immune challenged with bacterial LPS [10]. In this study, dietary supplementation with 500 mg/kg NAC was shown to improve the intestinal histological morphology of LPS-challenged newly weaned piglets by preventing LPS-mediated enhancement of crypt depth, reductions of villus height, and villus height to crypt depth ratio; all of which are indicators of potential intestinal absorption capacity and health. Furthermore, these authors also demonstrated that dietary NAC supplementation also alleviated LPS-mediated reductions of diamine oxidase (DAO) activity in the small intestinal mucosa and enhancement of DAO activity in the plasma; DAO is present in the intestinal mucosa, which is particularly abundant in rapidly dividing cells. DAO activity is a marker of intestinal mucosal maturation and integrity, as well as of mucosal injury and recovery, whereas plasma DAO provides an indication of the extent of mucosal injury. As such, it is evident that NAC is also capable of supporting mucosal barrier function under inflammatory conditions. As an antioxidant agent, NAC can also attenuate the adverse effects of intestinal oxidative stress caused by LPS. For example, Hao et al. (2021) also showed that dietary supplementation of NAC increased the activity of the endogenous antioxidants superoxide dismutase (SOD), catalase (CAT), and glutathione peroxidase (GSH-Px), ultimately enhancing the antioxidative capacity of the jejunal mucosa, where a significant decrease in malondialdehyde (MDA; biomarker of oxidative stress), H_2_O_2_, and O_2_^•−^ content were observed, along with increased content of GSH [92].

### 10.1. Swine Weaning Disorders

Early weaning stress is associated with both the generation of oxidative stress and changes in the gut microflora of piglets [93]. Factors contributing to post-weaning stress include hierarchy stress, new housing environment, and transition from liquid to solid feed, among many others [94]. Together, these factors negatively impact intestinal development and physiology and the gut microflora and host immunity, which collectively lead to reduced feed intake, poor growth performance, and increased disease susceptibility [92,94]. ROS, such as O_2_^•−^, H_2_O_2_, and OH, are potential toxic by-products for both aerobic and anaerobic gut microbes [93]. Furthermore, oxidative stress induced by weaning stress causes villus atrophy and suppresses the activities of digestive enzymes of weaned piglets [95]. At birth, the piglet gut microbiota is heavily influenced by the sow’s milk and is highly populated with lactic acid bacteria. However, the process of weaning significantly reduces the relative abundance of *Lactobacillus* spp., while simultaneously increasing the abundance of *Clostridium* spp., *Prevotella* spp., *Proteobacteriaceae,* and *Escherichia coli* and decreasing microbial diversity [94]. *E. coli*, an opportunistic enteric pathogen, is known to colonize the intestinal brush border and secrete enterotoxins that impair intestinal functions resulting in diarrhea [93]. The ability of pathogenic bacteria to utilize nutrients unusable to commensal bacteria further compounds pathogen overgrowth, intestinal inflammation, and post-weaning diarrhea [94].

NAC is known to protect cells against oxidative stress and suppress gut tissue damage [96]. In a study conducted by Xu et al. (2014), it was found that providing NAC in diets at a concentration of 500 mg/kg to weaned piglets led to a reduction of intestinal lipid peroxidation and ROS levels, while simultaneously restoring the activity level of endogenous antioxidant enzymes close to that of the normal suckling control group [93]. The reduction of ROS levels and increased activity of antioxidant enzymes observed in the Xu et al. study was thought to be attributed to improved gut redox status and diminished oxidative stress resulting from NAC’s direct and indirect antioxidant activities [93]. Furthermore, these authors also demonstrated that the NAC-containing diet altered the composition of the gut microbiota, where beneficial *Lactobacillus* and *Bifodobacterium* counts were increased, while *E. coli* counts were reduced. It appeared that *Lactobacillus* and *Bifodobacterium* counts are positively correlated with the activities of antioxidant enzymes and negatively correlated to MDA, H_2_O_2_, OH^•^, and NO in this study, whereas *E. coli* was positively correlated with ROS and negatively correlated with the activities of antioxidant enzymes in weaned piglets [93].

The presence of anti-nutritional factors (ANF) in solid feed is another area of concern around weaning, as ANF is known to induce oxidative stress. *β*-Conglycin (*β*-CG) is an ANF found in soybeans that causes inflammation, oxidative stress, mucosal barrier dysfunction, enterocyte damage, and diarrhea, and it impairs nutrient absorption in weaned piglets [11]. However, due to the high-quality source of protein content, soybeans are one of the main plant protein sources used in swine diets, despite the presence of *β*-CG [11]. While heating, pressurizing, fermenting, enzymatically hydrolyzing, and genetically modifying soybeans helps to reduce or inactivate *β*-CG, these practices are not able to completely mitigate the anti-nutritional properties of *β*-CG [11]. Given that *β*-CG induces oxidative stress, NAC may be beneficial in attenuating the adverse effects of *β*-CG in piglets. In a study conducted by Wang et al. (2021), dietary supplementation of NAC was found to numerically reduce the incidences of diarrhea in *β*-CG-challenged piglets, as well as concentrations of H_2_O_2_ (plasma and jejunum) and MDA (jejunum) [11]. Additionally, NAC supplementation to piglets was found to attenuate *β*-CG depleted enterocyte protein synthesis as evidenced by the increased abundance of intestinal fatty-acid binding protein (iFABP), as well as jejunal occludin and claudin-1 tight junction proteins [11]. Taken together, the administration of NAC may improve the intestinal integrity and function of piglets consuming ANF found in solid feed at weaning.

### 10.2. Porcine Epidemic Diarrhea (PED)

Porcine epidemic diarrhea virus (PEDV) is the causative agent of PED. PEDV infects the intestine of young pigs leading to acute, severe atrophic enteritis, profound diarrhea, vomiting, extensive dehydration, and high mortality (70–100%) in seronegative neonatal piglets [97]. PEDV infections have been found to increase certain plasma biochemical parameters, including ALT, total protein (TP), albumin (ALB), thyroglobulin (TG), blood urea nitrogen (BUN), chloride (CL), and gamma-glutamyl transferase (GGT) in pigs, which are indicative of systemic inflammation [7]. These authors found that dietary supplementation with NAC alleviated PEDV-induced injury to the small intestine and improved absorptive function, which was evident by the enhanced villus height and surface area, villus height-to-crypt depth ratio, decreased jejunal and ileal crypt depth, and increased plasma D-xylose concentrations, protein concentration, RNA/DNA ratios and protein/DNA ratios, and up-regulated I-FABP and villin expression in the small intestinal mucosa. Supplementation of NAC to PEDV-infected piglets also helped reduce oxidative stress as indicated by the reduction in H_2_O_2_ concentration in plasma and small intestinal mucosa.

Since the pig is often considered an ideal model for human biomedical research, because of their similar physiology [98], these porcine studies add further support to the reports in humans showing the immunoceutical potential of NAC in various species.

## 11. Cattle

Inflammation of the udder and teats (mastitis) and respiratory tract, as well as many postpartum reproductive disorders such as metritis and endometritis, are major challenges that the cattle industry continually faces as these are often complex multifactorial diseases [99,100,101]. Studies involving the use of NAC to treat bovine endometritis and mastitis have shown promising results. For example, in a study conducted by Constantin and Șonea (2018), in which NAC was used to treat bovine endometritis, the clinical cure rate in the NAC group was 77.2% versus 43.4% in the non-NAC group [102]. Furthermore, the NAC group also presented with a higher pregnancy rate of 66.7% versus 54.6% in the non-NAC group. In a study conducted by Yang et al. (2016), NAC was shown to be an important modulator of antibiotic activity against the major bovine mastitis pathogens, including *Staphylococcus aureus*, *Streptococcus dysgalactiae*, *E. coli,* and *Streptococcus agalactiae*; the addition of 10 mM of NAC reduced the minimum inhibitory concentrations (MIC) of penicillin and ampicillin but led to the enhancement of erythromycin and ciprofloxacin’s MIC for all tested bacterial strains [9].

Bovine respiratory disease complex (BRD) is a multifactorial disease encompassing a wide range of both viral and bacterial infections, and it remains the major cause of morbidity and death in feedlot cattle [103,104] and veal calves [105]. Decreased immune defenses caused by stressors, including viral pathogens, make cattle more susceptible to infection by existing pathogenic and opportunistic bacteria in the upper respiratory tract [104,106]. Interestingly, studies involving the use of NAC to prevent or attenuate BRD are lacking, but an in vitro study conducted by Lin et al. (2020) revealed that NAC can attenuate apoptosis and autophagy in lung cells, which might be beneficial in cattle, warranting further research [3].

## 12. Poultry

Several studies have also studied the use of NAC in poultry production, particularly its use in addressing problems caused by aflatoxin B_1_ (AFB_1_) intoxication and heat and cold stress. In a study conducted by Valdivia et al. (2001), their results suggested that NAC supplementation (800 mg NAC/kg BW per day) helped to mitigate the severity of AFB_1_ toxicity as evidenced by the protection against AFB_1_-mediated reductions in body weight and liver and renal damage, as well as AFB_1_-induced biochemical alterations [107]. In a study conducted by Li et al. (2020), it was found that the supplementation with 0.1% NAC mitigated cold-induced oxidative stress in broilers by increasing the activities of hepatic antioxidant enzymes [108]. Similarly, Yi et al. (2016) demonstrated that dietary supplementation of 1 g/kg of NAC was able to improve the growth performance of heat-stressed broilers, intestinal morphology, and absorptive function, maintain intestinal energy metabolism, and mitigate intestinal oxidative stress [109].

Given the findings of the above studies, NAC appears to be a promising low-cost and safe therapeutic agent that could be more widely used in the livestock industry to address issues and diseases costing the livestock industry millions each year.

## 13. Companion Animals

### 13.1. Dogs or Cats

#### 13.1.1. Acetaminophen Toxicosis

Acetaminophen toxicosis is among the 10 most common toxicoses in dogs based on the number of calls that were received at the ASPCA Animal Poison Control Center between 2001 and 2005 [110]. Cats are especially sensitive to the toxic effects of acetaminophen due to a deficiency in a specific high-affinity acetaminophen glucuronyl transferase [111]. Acetaminophen toxicity can result from either a single toxic dose or from repeated cumulative dosages resulting in methemoglobinemia, hepatotoxicosis, facial and paw edema, depression, weakness, tachypnea, dyspnea, cyanosis, icterus, vomiting, hypothermia, hepatic necrosis, and death in severe cases [112,113]. Clinical signs of toxicosis are not observed in dogs below doses of 100 mg/kg, and acetaminophen is used therapeutically in dogs at doses of 10 mg/kg every 12 h [112]. Hepatotoxicity in dogs is possible at doses that exceed 100 mg/kg, and at 200 mg/kg, methemoglobinemia is possible [112]. Unlike dogs, there is no safe acetaminophen dose for cats [114]. Signs of toxicity have been found at doses as low as 10 mg/kg [115].

Acetaminophen is primarily metabolized as glucuronide and sulfate conjugates in dogs and cats [112]. Due to a deficiency in the hepatic enzyme, glucuronyl transferases, cats form glucuronides with compounds slowly or not at all [116]. As a result of possessing relatively few isoforms of the specific high-affinity acetaminophen glucuronyl transferase, which mediates the conjugation of acetaminophen with glucuronic acid resulting in its elimination, more of the drug is conjugated to sulfates [111]. However, the sulfation pathway also has a finite capacity, which is much lower in cats compared to other species [111]. Once the glucuronide conjugation and sulfation pathways are saturated, excess acetaminophen is oxidized via the cytochrome P450 microsomal enzyme, resulting in the formation of NAPQI, a highly toxic metabolite [111]. Under normal circumstances, NAPQI is inactivated following its conjugation with GSH; however, in acetaminophen toxicosis, GSH stores are rapidly depleted [116]. If inactivated, NAPQI can cause necrosis of hepatic tissue, as well as covalently bind to cellular macromolecules, mediating the conversion of hemoglobin to methemoglobin and inducing the formation of Heinz body [117].

As with humans, the antidote of choice for acetaminophen poisoning in dogs and cats is NAC [110]. NAC works by directly binding to acetaminophen metabolites thereby rendering them inactive and serving as a GSH precursor [118,119]. NAC can also reduce the extent of hepatic injury and methemoglobinemia [118,119]. To treat acetaminophen toxicosis, activated charcoal is first given to absorb acetaminophen [112]. Oral administration of NAC is then given two to three hours following activated charcoal administration, as activated charcoal may absorb NAC if given too early thereby reducing its effectiveness [112]. A 5% NAC solution is administered orally at an initial loading dose of 140 mg/kg, followed by 70 mg/kg every six hours for at least seven doses [120]. Due to its pungent aroma, oral administration of NAC typically causes nausea and vomiting in dogs and cats [121]. While NAC is not labeled for intravenous use, NAC can be given intravenously slowly in life-threatening situations at an initial dose of 140 mg/kg, followed by a maintenance rate of 70 mg/kg every six hours for seven treatments [122]. Each sterile dose is infused over a period of 30 to 60 min through a 0.2 m Millipore filter [122]. It is advised that when NAC is administered intravenously, doses should be given slowly to minimize potential adverse reactions (hypotension, bronchospasm, and flushing) [121].

#### 13.1.2. Infectious Keratitis

Infectious keratitis is the most common ocular disease presented as corneal ulceration (ulcerative keratitis) in dogs in cats [123,124]. Corneal ulcers are frequently the result of trauma and are not always primarily infected; however, they can be rapidly contaminated with bacteria [123,124,125]. Diagnosis and management of infectious keratitis cases are crucial as they are a potential threat to sight [126,127,128]. The three most common bacterial organisms responsible for ulcerative keratitis in dogs and cats are *Staphylococcus*, *Pseudomonas,* and *Streptococcus* species [129,130,131,132,133,134,135].

Treatment of infectious keratitis involves topical antimicrobial treatment [136]. Due to the wide spectra of activity between antibiotics and the various bacterial strains involved, there is currently no antimicrobial agent available that is effective against all associated pathogens [136]. While some authors advise the use of a combination of different antimicrobial agents to treat this condition, this can potentially lead to reduced efficacy [136,137,138,139]. Many antibiotics are known to interfere with each other or even have antagonistic effects [139]. Furthermore, repeated exposure to antibiotics may alter the bacterial ocular flora composition and favor the colonization of pathogenic bacteria [140]. Normal conjunctival flora has long been suggested to play a crucial role in ocular defense against invasive infections by inhibiting the colonization of pathogenic species [141]. The prognosis for infectious ulcerative keratitis is guarded, with up to 57% of patients requiring surgical interventions even with intensive antimicrobial therapy [142]. Furthermore, with the increasing development of bacterial resistance, there is a need for an alternative substance with antimicrobial properties.

Various studies have demonstrated that NAC possesses antimicrobial activities and is able to disrupt the biofilm formation of different bacterial species in various anatomical sites [143,144,145,146,147,148]. The bacterial microorganisms associated with infectious ulcerative keratitis in dogs, cats, and humans are also known to form biofilms [149,150,151,152]. While NAC is commonly used topically in human ophthalmology to treat corneal wounds, chemical injuries, keratitis, dry eye disease, and meibomian gland dysfunction, studies investigating the effect of NAC on pathogenic bacterial strains causing corneal ulceration in dogs and cats are lacking [136,153]. The study conducted by Walter et al. (2023) appears to be the first study to be conducted to determine the in vitro antimicrobial activity of NAC against common pathogens associated with infectious keratitis in dogs and cats [136]. NAC was observed to have an in vitro antimicrobial effect against all 38 bacterial isolates tested at relatively low concentrations (0.156–0.625%, 1.56–6.25 mg/mL) [136]. Furthermore, all methicillin-resistant *S. pseudintermedius* isolates that were tested in the study were found to be susceptible to 0.312% NAC [136]. Future research is needed to investigate the antimicrobial effect of NAC in vivo in infectious keratitis patients, as in vitro studies do not address factors such as the expected contact time of isolates with NAC when it is applied to the ocular surface. Topical ophthalmic therapeutics typically remain on the ocular surface for 5–10 min in dogs and cats before they are cleared through the nasolacrimal duct and spilled over the lower eyelid [154]. Furthermore, dilution of NAC due to tear production is also not accounted for in in vitro studies. Nevertheless, NAC appears to be a promising antimicrobial agent that can reduce or replace the use of topical antibiotics for the treatment of infectious ulcerative keratitis.

#### 13.1.3. Type 1 Diabetes Mellitus

Canine diabetes, like human diabetes, is divided into two types: type 1 diabetes and type 2 diabetes, with the former being insulin-dependent and the latter being non-insulin-dependent [155]. Most canine diabetes cases are type 1 diabetes mellitus, which is a metabolic disease associated with insulin deficiency and often hypercholesterolemia [155,156]. Clinical signs of diabetes in dogs include polydipsia, polyuria, and weight loss [155]. Disease progression is often associated with further complications that can cause multiple organ damage, such as pancreatitis, kidney failure, motor dysfunction, cardiovascular disease, cataracts, hypercholesterolemia, digestive system diseases, and stroke [157]. Furthermore, diabetes has been shown to be closely linked to atherosclerosis due to the exacerbation of inflammatory processes and stimulation of the formation of new blood vessels [158]. Oxidative stress is also involved in the pathogenesis of diabetes mellitus [159].

Several studies have been conducted on the use of NAC in the treatment of canine diabetes mellitus. In the study conducted by Wang et al. (2023), the combination of NAC with insulin in the treatment of dogs with type 1 diabetes was found to be able to stably maintain blood glucose levels within the normal range, slow down the rate of weight loss, effectively reduce liver injury, and correct cholesterol metabolism disorder and thus effectively prevent the development of hypercholesterolemia [155]. Ma et al. (2023) reported similar findings in their study investigating the protective mechanism of NAC in combination with insulin against renal injury in diabetic dogs [160]. The authors reported that the combination of insulin with NAC was able to attenuate renal injury in type 1 diabetic dogs by regulating mitochondrial dynamics and FUNDC1-mediated mitophagy [160]. Huo et al. (2022) demonstrated that the combination of NAC with insulin relieved diabetes mellitus-induced inflammation and pyroptosis hepatic injury via the NLRP3/NF-κB pathway [161]. Overall, the combination of NAC with insulin appears promising in the treatment of canine type 1 diabetes mellitus.

#### 13.1.4. Parvovirus

Canine parvovirus (CPV) is a highly contagious virus that affects both domestic dogs and wild canids worldwide [162]. While CPV infection can affect all ages, severe infection is most common in puppies between the ages of 6 weeks and 4 months, with higher incidences in animal shelters, pet stores, and breeding kennels [162,163]. CPV affects the gastrointestinal tracts of dogs, preferentially infecting and destroying the rapidly dividing cells of the small-intestinal crypt epithelium [164]. Clinical signs of CPV infection include anorexia, lethargy, vomiting, often hemorrhagic diarrhea, abdominal pain and bloating, and hypothermia, with most deaths occurring within the first 48 to 72 hours following the onset of clinical signs [165]. Due to the lack of effective antiviral therapy, supportive therapy is the only option available [162].

In recent years, oxidative stress was observed to be associated with parvovirus infection, with marked enhancement of reactive oxygen and nitrogenous species, lipid peroxidation, DNA damage, and low antioxidant reserve parvo canine patients [166,167,168]. With oxidative stress being implicated in the pathogenesis of viral diseases, such as feline coronavirus [169], bovine herpes-virus-1 [170], porcine reproductive and respiratory syndrome [171], and rotavirus [172], emphasis has been given on the use of antioxidants for the management of viral diseases [173,174,175]. Thus, the incorporation of NAC into the therapeutic regimen against CPV may help to ameliorate the clinical signs of CPV. Indeed, in the study conducted by Gaykwad et al. (2018), NAC treatment of parvo-infected dogs was found to progressively improve the leukocyte, neutrophil, monocyte, and eosinophil counts over time in comparison to parvo-infected dogs that only received supportive treatment [162]. Additionally, NAC treatment was found to significantly improve glutathione S-transferase (GST) activity, as well as decrease nitric oxide and MDA concentrations in plasma on day 3 and day 5 following initiation of treatment compared to the group that only received supportive treatment. The authors evaluated oxidative stress on the basis of GST activity and nitric oxide and MDA concentration in plasma. Chethan et al. (2023) also reported similar results with markedly reduced concentrations of MDA, nitric oxide, and IFABP-2 in CPV-positive dogs supplemented with NAC, resveratrol, and ascorbic acid compared to the control group, which only received supportive therapy [176]. Supplementation with NAC and resveratrol was also found to markedly improve total leukocyte and neutrophil count in CPV-affected dogs. The findings from these studies suggest that NAC represents a potential additional treatment option that should be considered when treating CPV canine patients.

#### 13.1.5. Otitis Externa

Canine otitis externa is the most common, often chronic, disorder affecting the ear canal of dogs and is associated with a high rate of recurrence [177]. In an epidemiological study involving 2012 dogs conducted in 2017, the frequency of otitis externa diagnosis was 15.9%, with a recurrence rate of 24% [178]. Underlying allergic conditions, such as atopic dermatitis or cutaneous adverse food reactions, are often the primary cause of otitis, with secondary bacterial otitis as the complicating perpetuating factor [177,179]. Common bacterial pathogens associated with canine otitis externa include *Staphylococcus pseudintermedius*, *Pseudomonas aeruginosa*, *β*-haemolytic *Streptococcus* spp., and *Proteus* spp. Currently, commercially available treatments approved to treat otitis externa are limited in variety as the antibacterial agents present in the products are from a limited number of drug classes [179]. Furthermore, these commercial products often contain ototoxic ingredients, such as aminoglycosides, that can result in temporary or permanent hearing loss in dogs [180,181].

NAC’s antimicrobial and mucolytic properties, as well as its ability to disrupt biofilm formation, make it a promising potential alternative for the treatment of otitis externa in dogs. Indeed, in an in vitro study conducted by May et al. (2016), NAC was found to have antimicrobial activity against all twenty-two isolates from canine clinical cases of otitis externa [179]. NAC’s minimum inhibitory concentration (MIC) for all tested isolates ranged from 5 to 20 mg/mL. These findings are further corroborated by Son and Bae (2021) and Chan et al. (2019) [143,182]. According to Son and Bae (2021), NAC alone was found to be effective at inhibiting *P. aeruginosa*, which was frequently isolated from canine otitis externa cases [182]. In the study conducted by Chan et al. (2019), NAC was found to be effective against all 110 bacterial and yeast isolates obtained from otitis externa cases with MICs ranging from 2500 to 10,000 g/mL [143]. Studies have also been conducted on the use of NAC in combination with antimicrobials on common canine otitis externa bacterial isolates [177,182]. It appears that NAC interactions with antimicrobials when used against otitis externa bacterial isolates are often indifferent or antagonistic rather than synergistic [177,182].

## 14. NAC Molecular Mechanisms of Action (MOA)

**Figure 1:**
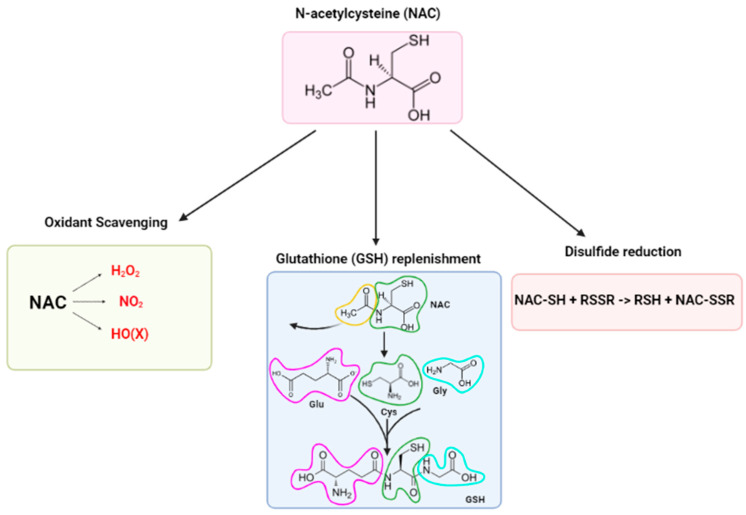
The three major narratives of NAC’s mechanisms of action.

Despite the vast number of NAC-related publications over the recent decades, a clear MOA and a consensus explanation for NAC’s antioxidant and radical scavenging activities remains unclear [16,17,37]. Three major narratives have been proposed to explain the observed effects of NAC: (1) oxidant scavenger, (2) GSH replenishment, and (3) disulfide reductant (Figure 1). However, according to Pedre et al. (2021) and Ezeriņa et al. (2018), these narratives are only applicable under very specific circumstances [16,17]. An alternative MOA is also slowly emerging, involving (4) the sulfane sulfur branch of the NAC metabolism [17]; these per- and polysulfides possess antioxidant and cytoprotective properties, which may explain the observed effects attributed to the NAC [17].

(1) Direct antioxidant activity of NAC as an oxygen radical scavenger

For a compound to act as an antioxidant in a biological matrix, its reaction rate with oxidants must be higher than that of endogenous antioxidants and much higher than that of the substrates present [37]. Additionally, the location of ROS generation, the type of ROS produced, and the relative concentration of endogenous antioxidants at the location site must also be taken into consideration when determining the ability of an antioxidant to exert its antioxidant activities [28]. Table 3 provides the reaction rate constants of NAC and other endogenous enzymatic antioxidants with primary oxidant species.

Under physiological conditions, the reaction rate constant of NAC is consistently lower than that of other endogenous enzymatic and non-enzymatic antioxidants, including GSH, cysteine (Cys), and peroxiredoxins [1,37]. Consequently, NAC reactions with primary oxidant species are relatively slow when considering that the concentrations of substrates and endogenous antioxidants are much higher than the concentration of NAC [1,37]. In certain cases, some oxidant species are not targeted by NAC, since the rate of reaction is much too slow to be plausible [37]. These oxidant species include H_2_O_2_, O_2_^•−^, OHNOO, and HO^•^ [37]. However, in situations where the concentration of NAC is higher than that of other thiols (i.e., GSH, Cys), NAC can potentially act on the oxidant species NO_2_ and hypohalous acids (HOX) [1,37]. In the case of HOX, NAC can act on HOX because its concentration is higher than that of GSH and Cys in the locality [37]. This situation can be brought on by either pathological conditions or exposure to environmental stressors such as exposure of lung fluids to an inflammatory or oxidative process [37]. Similarly, NAC can also potentially act on NO_2_, which is a major component of both indoor and outdoor air pollution and is damaging to the lung epithelium [37].

(2) Indirect antioxidant activity of NAC via glutathione replenishment

In addition to NAC’s direct antioxidant activity, NAC also exerts an indirect antioxidant effect through its ability to replenish depleted GSH stores [37]. Most of the antioxidant effects attributed to NAC are the result of increased intracellular GSH [28]. Consequently, certain conditions must be satisfied for NAC to exert its antioxidant activity [28]; the first condition being that the enzymatic machinery required for GSH synthesis is non-defective and expressed at adequate levels, and the second being that GSH levels must be depleted in order for NAC to confer any beneficial effect [28]. Indeed, as demonstrated by Giustarini et al. (2012), neither short-term (5 min) nor long-term (2 weeks) administration of NAC resulted in the elevation of GSH levels in the healthy organs of rats [25]. The lack of elevation in GSH levels under normal conditions is due to a negative feedback mechanism embedded in the GSH biosynthesis pathway [17].

GSH’s poor bioavailability and limited ability to cross phospholipid bilayers make the administration of GSH suboptimal [28]. Similarly, Cys undergoes rapid oxidation to its disulfide moiety upon delivery, thereby generating an inactive disulfide cystine (Cys-Cys), and due to its poor solubility, the sulfhydryl functional group on Cys is rendered temporarily inaccessible [28]. However, acetylating the N-terminal end of Cys, thus creating the compound NAC, increases the stability of the molecule and allows for more efficient delivery of reduced sulfhydryl moieties [28]. While the exact mechanism of how NAC delivers Cys remains unclear, it is postulated that when free intracellular reduced Cys is required for GSH synthesis, intact NAC will permeate the cell membrane before undergoing hydrolysis to yield Cys [28]. The deacetylation of N-acetyl-L-amino acids is catalyzed by aminoacylases I, II, and III [192]; cytosolic acylase I is the aminoacylase responsible for the deacetylation of NAC [37]. Determination of the activity and presence of cytosolic acylase I in various organs of several mammalian species (rat, rabbit, dog, monkey, and man) was carried out by Yamauchi et al. (2002), who concluded that acylase activity was the highest in the kidney of all species studied [193]. These authors found that hepatic cytosolic acylase I activity was 10–22% of that in the kidneys of the rat, rabbit, monkey, and man; however, liver acylase activity in the dog was negligible. Based on these results, the kidney and liver appear to be the main organs responsible for the biotransformation of NAC to the amino acid cysteine in mammals.

(3) NAC as a disulfide reductant

Through the thiol-disulfide interchange mechanism, NAC acts as an efficient reducing agent of protein disulfides [37]. Protein disulfides serve as inter- and intra-subunit crosslinks in secondary and tertiary protein structures, and, as such, play a critical role in maintaining the structure of many proteins, including mucus proteins [194]. Disulfides are also produced through thiol oxidation; a process involved in defense mechanisms against oxidative stress and in redox regulation of cell signaling [194].

The classical thiol-disulfide interchange reaction involves a nucleophilic substitution (S_N_2) of a thiol in disulfides with another thiol [194]. This S_N_2-type nucleophilic substitution mechanism is a one-step reaction, whereby, in the case of NAC, the thiolate in NAC binds to the central sulfur of the disulfide, thus breaking the disulfide bond and the leaving thiol released via a trisulfide-like transition state structure [37,194]. The rate of the thiol-disulfide interchange reaction is dependent on the nucleophilicity of the thiolate; the higher the nucleophilicity, the greater the reducing ability [37]. The order of S nucleophilicity of the NAC, GSH, and Cys thiols is NAC > GSH > Cys, and, therefore, in comparison to GSH and Cys, NAC has the greatest disulfide-reducing ability [37].

NAC’s disulfide-reducing ability is responsible for its mucolytic activity, and, as such, NAC is also considered a mucolytic drug. Mucolytic drugs act on the mucus layer lining the respiratory tract to increase mucosal clearance by reducing the heavily cross-linked mucin polymers, which are the major macromolecular constituents of mucus that are characterized by cysteine-rich domains in their N and C termini [37]. The N and C termini mediate the extension of mucin polymers through end-to-end disulfide linkage of mucin monomers [37]. Mucins also contain Cys-rich regions in their internal domains that are prone to forming internal cross-links when oxidized [37]. Under normal physiological conditions, airway mucus gels are lightly cross-linked, which allows for easy transportation up the mucociliary escalator towards the laryngopharynx, where mucus can be swallowed down the esophagus [37]. However, under pathological conditions involving inflammation and oxidative bursts leading to the oxidation of internal Cys thiols, disulfide cross-links are generated between internal Cys domains rendering the mucus to be heavily crosslinked [37]. Heavily cross-linked mucus cannot be easily transported by the mucociliary escalator, and, consequently, mucus accumulation results in airflow obstruction, atelectasis (collapsed lung), and renders the lungs susceptible to microbial infection [37].

## 15. Alternative MOA via the Sulfane Sulfur Branch of NAC Metabolism

The use of NAC to replenish GSH levels and protect against GSH-depleting xenobiotics is well-supported by the literature and has, therefore, led many studies to conclude that the restoration of GSH levels by NAC is the cause of the observed beneficial health outcome in all situations [17]. However, if this were true, the observed beneficial effect of NAC should disappear when *γ*-glutamylcysteine ligase, which catalyzes the first step in the GSH biosynthesis pathway, is inhibited by buthionine sulfoximine (BSO) [17]; in the presence of BSO, NAC should not be able to restore GSH levels and, therefore, should not be able to exert any beneficial effects. However, several studies have also observed beneficial effects of NAC despite GSH replenishment being blocked by BSO [195,196,197,198,199]. These findings suggest that there is an alternative mechanism through which NAC exerts its cytoprotective activities that is independent of GSH replenishment.

In recent years, studies have shown that the exposure of cells to NAC results in enhanced endogenous H_2_S production [16,200,201,202]. Elevated H_2_S levels within the physiological range (up to ~20 nM) have been linked to a range of cytoprotective effects [203]. At the organism level, H_2_S acts as a positive regulator of vasoactivity and provides protection against ischemia-reperfusion injury, while at the cellular level, H_2_S releasing agents inhibit apoptosis, enhance mitochondrial bioenergetics, and provide protection against oxidative stress [204,205,206,207,208]. While the exact molecular mechanism by which H_2_S exerts its beneficial effects is not yet well understood, three hypotheses have been proposed to explain the observed persulfide-mediated cytoprotection [17]. For the purposes of this review, only the third hypothesis will be discussed, as it provides a link between H_2_S and the protective effects of NAC. This hypothesis argues that the observed beneficial effects of H_2_S may be the result of sulfane sulfur species, particularly per-(RSSH) and poly-(RSS_n_SR) sulfides, which are products of H_2_S oxidation [17]. Sulfane sulfur species are mainly generated by sulfide quinone oxidoreductase (SQR), which catalyzes the first step in the metabolism of H_2_S to yield a sulfane sulfur metabolite. Persulfides can also be generated through the oxidation of H_2_S by cytochrome C or as products of a non-enzymatic process, whereby H_2_S reacts with sulfenic acids, which can also be formed under conditions of oxidative stress [17]. Sulfane sulfur species are known to have cytoprotective properties and have been shown to provide protection against the damaging effects of electrophiles [209,210,211], metals [212], oxidants [16], and cyanide [213,214]. This hypothesis suggests that persulfides act as scavengers of one/two-electron oxidants and electrophiles due to their higher reactivity in comparison to corresponding thiols [215,216,217]. This higher reactivity can be explained by the ∝-effect and the lower pK_a_ of persulfides in comparison to corresponding thiols [17]; the ∝-effect refers to the increased nucleophilicity of an atom due to the presence of an adjacent atom with lone pair electrons [17]. In the case of persulfides, the nucleophilicity of the adjacent persulfide’s outer sulfur atom is enhanced due to the presence of the lone pair electrons of the inner sulfur atom [17]. Additionally, persulfides’ lower pK_a_ value increases the proportion of its reactive deprotonated form at physiological pH [202,215]. Persulfides have also been suggested to be highly efficient terminators of radical chain reactions despite the lack of data on the interaction between persulfides and ROS [218].

To summarize, multiple lines of evidence exist to support the alternative narrative that NAC acts as a donor of sulfane sulfur species, which, in turn, exerts cytoprotective effects and enhances cellular reducing capacity. While the disulfide-breaking agent and oxidant scavenger narratives can be used to explain health benefits observed following the restoration of depleted GSH stores by NAC, this only applies to situations characterized by severe GSH depletion, and not to situations independent of GSH biosynthesis. In contrast, the H_2_S/sulfane sulfur narrative explains the antioxidative properties of NAC in a GSH-independent manner. Sulfane sulfur species derived from NAC enhance resistance to oxidative stress by modulating protein activity, protecting thiols against oxidative damage and scavenging radicals. This alternative narrative reconciles the antioxidative properties of NAC with its inherent inefficiency as a direct antioxidant. Thus, the discovery that NAC can also exert its antioxidative effects in a GSH-independent manner warrants re-evaluation of the possible therapeutic use of NAC regarding other pathological conditions unrelated to disturbances in GSH levels. Additional work is, therefore, required to further determine the conditions by which NAC produces sustained low-level H_2_S/sulfane sulfur production and to which extent this confers therapeutic effects.

## 16. Conclusions

NAC’s antioxidant and cytoprotective properties make it a promising therapeutic agent for a wide range of conditions in which oxidative stress plays a major role in the onset and progression of the disease. Numerous studies have been conducted on the use of NAC for treating human pathological conditions, ranging from pulmonary and metabolic diseases, and infectious pathogens such as SARS-CoV2. NAC has also been proven beneficial in treating and alleviating ailments of domesticated animals; especially porcine disorders associated with weaning.

Due to NAC’s use for treating a wide range of conditions, further studies are required to determine adequate dosages, appropriate routes of administration, and treatment protocols for each condition to ensure that NAC efficiently exerts its antioxidant, cytoprotective, anti-inflammatory, and mucolytic properties. Special consideration must also be given to identifying any cross-reactivity with specific compounds that, when combined with NAC, can cause it to act as a prooxidant, a substance that induces oxidative stress. For example, Solov’eva et al. (2007) recently discovered that the simultaneous addition of NAC with vitamin B_12b_ (hydroxocobalamin) to cell culture produced a cytotoxic effect [219]. Future potential applications of NAC could include its use for the treatment of respiratory diseases across species, including livestock, where respiratory disease is one of the most common and costly factors affecting the livestock industry, particularly the swine, beef, and dairy cattle industries. Respiratory diseases commonly affect these animals and other species during the critical post-weaning period and can have long-lasting effects on health and productivity. NAC’s mucolytic, antioxidant, antimicrobial, and anti-inflammatory properties should prove useful in treating and attenuating the negative impacts associated with respiratory disease and other inflammatory conditions encountered in both human and veterinary medicine.

## Figures and Tables

**Table 3:** Reaction rate constants of N-acetylcysteine (NAC), cysteine (Cys), and glutathione (GSH) towards the oxidant species H_2_O_2_, O_2_^•−^, HO^•^, HO(X), and NO_2._

Oxidant	Antioxidant	K (M^−1^ s^−1^)	References
H_2_O_2_	NAC	0.16	[183]
	GSH	0.89	[183]
	Cys	2.9	[183]
O_2_^•−^	NAC	68	[184]
	GSH	200	[185]
	Cys	15	[184]
HO^•^	NAC	1.36 × 10^10^	[186]
	GSH	1.64 × 10^10^	[187]
	Cys	5.35 ± 0.2 × 10^9^	[188]
HO(X)	NAC	0.29 ± 0.04 × 10^8^	[189]
	GSH	1.2 ± 0.2 × 10^8^	[189]
	Cys	3.6 ± 0.5 × 10^8^	[189]
NO_2_	NAC	1 × 10^7^	estimated by [190]
	GSH	2 × 10^7^	[191]
	Cys	6 × 10^7^	[191]
